# Oxidative Stress in Human Aorta of Patients with Advanced Aortoiliac Occlusive Disease

**DOI:** 10.5935/1678-9741.20160086

**Published:** 2016

**Authors:** Márcio Luís Lucas, Cristina Campos Carraro, Adriane Belló-Klein, Antonio Nocchi Kalil, Newton Aerts

**Affiliations:** 1 Santa Casa de Porto Alegre, Porto Alegre, RS, Brazil.; 2 Universidade Federal do Rio Grande do Sul (UFRGS), Porto Alegre, RS, Brazil.; 3 Universidade Federal de Ciências da Saúde (UFCSPA), Porto Alegre, RS, Brazil.

**Keywords:** Oxidative Stress, Arterial Occlusive Diseases, Reactive Oxygen Species

## Abstract

**Introduction:**

Oxidative stress seems to be a role in the atherosclerosis process, but research in human beings is scarce.

**Objective:**

To evaluate the role of oxidative stress on human aortas of patients submitted to surgical treatment for advanced aortoiliac occlusive disease.

**Methods:**

Twenty-six patients were divided into three groups: control group (n=10) formed by cadaveric organ donors; severe aortoiliac stenosis group (patients with severe aortoiliac stenosis; n=9); and total aortoiliac occlusion group (patients with chronic total aortoiliac occlusion; n=7). We evaluated the reactive oxygen species concentration, nicotinamide adenine dinucleotide phosphate-oxidase, superoxide dismutase and catalase activities as well as nitrite levels in samples of aortas harvested during aortofemoral bypass for treatment of advanced aortoiliac occlusive disease.

**Results:**

We observed a higher level of reactive oxygen species in total aortoiliac occlusion group (48.3±9.56 pmol/mg protein) when compared to severe aortoiliac stenosis (33.5±7.4 pmol/mg protein) and control (4.91±0.8 pmol/mg protein) groups (*P*<0.05). Nicotinamide adenine dinucleotide phosphate oxidase activity was also higher in total aortoiliac occlusion group when compared to the control group (3.81±1.7 *versus* 1.05±0.31 µmol/min.mg protein; *P*<0.05). Furthermore, superoxide dismutase and catalase activities were significantly higher in the severe aortoiliac stenosis and total aortoiliac occlusion groups when compared to the control cases (*P*<0.05). Nitrite concentration was smaller in the severe aortoiliac stenosis group in comparing to the other groups.

**Conclusion:**

Our results indicated an increase of reactive oxygen species levels and nicotinamide adenine dinucleotide phosphate-oxidase activity in human aortic samples of patients with advanced aortoiliac occlusive disease. The increase of antioxidant enzymes activities may be due to a compensative phenomenon to reactive oxygen species production mediated by nicotinamide adenine dinucleotide phosphate oxidase. This preliminary study offers us a more comprehensive knowledge about the role of oxidative stress in advanced aortoiliac occlusive disease in human beings.

**Table:** 

**Abbreviations, acronyms & symbols**	
AAA	= Abdominal aortic aneurysm
AOD	= Aortoiliac occlusive disease
BMI	= Body mass index
CAT	= Catalase
DCF	= 2,7-dichlorofluorescein
H_2_O_2_	= Hydrogen peroxide
ICU	= Intensive care unit
LVEF	= Left ventricular ejection fraction
NADPH	= Nicotinamide-adenine-dinucleotide-phosphate
NO	= Nitric oxide
NOS	= Nitric oxide synthase
O_2_-	= Superoxide anion
OH-	= Hydroxyl radical
ONOO-	= Peroxynitrite
ROS	= Reactive oxygen species
SAS	= Severe aortoiliac stenosis
SOD	= Superoxide dismutase
TAO	= Total aortoiliac occlusion

## INTRODUCTION

Aortoiliac occlusive disease (AOD) provoked by atherosclerosis is an important cause of morbidity and mortality worldwide, which begins at the aortic terminus and common iliac artery origins and slowly progresses proximally and distally over time, resulting in a severe aortoiliac stenosis (SAS) or in a total aortoiliac occlusion (TAO)^[^^1^^]^. Although advances in the endovascular techniques, aortobifemoral bypass is still the gold standard treatment for patients with SAS and TAO^[^^1^^,^^2^^]^.

The cellular and molecular basis for atherosclerosis is complex and is not completely understood^[^^3^^]^. It is widely accepted that oxidative stress plays important role in the pathogenesis of atherosclerosis^[^^4^^]^. Oxidative stress is caused by an imbalance between the production of reactive oxygen species (ROS) and the antioxidant capacity of the biological system^[^^5^^]^. ROS such as superoxide anion (O_2_^-^), hydroxyl radical (OH^-^), and hydrogen peroxide (H_2_O_2_), are produced through different pathways, but the major source of ROS in the vasculature is nicotinamide-adenine-dinucleotide-phosphate (NADPH) oxidase^[^^5^^,^^6^^]^. Superoxide dismutase (SOD) is the major cellular defense system to remove O_2_^-^ in vascular cells by converting O_2_^-^ into H_2_O_2_, while catalase (CAT) converts two molecules of H_2_O_2_ into water and oxygen^[^^7^^]^. Nitric oxide (NO) - synthetized by nitric oxide synthase (NOS) - is a multifactorial free radical that plays a key role in the physiological regulation of the cardiovascular system, and changes in its production and/or bioavailability follow or even precede diseases such as atherosclerosis^[^^8^^]^. Superoxide anion may react with NO to form peroxynitrite (ONOO^-^), resulting in an increased cellular damage^7^. Moreover, inactivation of NO by O_2_^-^ limits the NO bioavailability leading to increased vasoconstriction, as commonly observed in the progression of atherosclerosis^[^^7^^]^.

Some experimental studies have investigated the role of oxidative stress in the atherosclerosis process^[^^4^^,^^9^^,^^10^^]^, but research in human beings are rare^[^^11^^]^. In this study, we evaluated the role of oxidative stress in patients's aortas whith SAS and TAO submitted to aortobifemoral bypass. Thus, we proposed to compare the oxidative damage in different degrees of AOD in human beings.

## METHODS

The project was approved by the Research Ethics Committee of Santa Casa de Porto Alegre and all patients signed free and informed consent forms. We reviewed medical records for patients with SAS and TAO electively submitted to aortobifemoral bypass by the first author as previously described^[^^2^^]^.

All the patients were submitted to surgical procedure due to a limiting claudication or critical limb ischemia (rest pain and/or non-healing wound). Patients with acute aortoiliac occlusion, abdominal aortic aneurysm (AAA) thrombosis, and submitted to previous aortoiliac intervention (endovascular or open repair) were excluded. Data were collected on age, gender, comorbidities and clinical presentation for patients with SAS or TAO. Laboratory profiles and surgical data were also routinely collected. All the patients were submitted to computed tomography angiography to plan the surgical procedures.

Human specimens of infrarenal abdominal aorta were removed in the operating room during the proximal anastomosis of aortobifemoral bypasses. After removing the calcifications, the aortic samples were immediately stored at -70°C for further analysis of oxidative stress parameters. They were macerate in liquid nitrogen. After that, it was homogenized (KCl 150 mmol/L; phenyl-methyl-fluoro-sulfonyl 20 mmol/L, 1:100) in the Ultra-Turrax homogenizer. Posteriorly, it was performed a sonification with the Hielscher Ultrasound Technology device^[^^12^^]^.

ROS concentration was measured by DCFH-DA fluorescence emission (Sigma-Aldrich, USA). Dichlorofluorescein diacetate is membrane permeable and is rapidly oxidized to the highly fluorescent 2,7-dichlorofluorescein (DCF) in the presence of intracellular ROS. The samples were excited at 488 nm and emission was collected with a 525 nm long pass filter. It was expressed as nmols per milligram of protein^[^^13^^]^.

Measurement of NADPH oxidase activity was assayed with spectrophotometric method^[^^14^^]^. SOD activity, expressed as U/mg protein, was based on the inhibition of superoxide radical reaction with pyrogallol^[^^15^^]^. CAT activity was determined by following the decrease in 240 nm absorption of hydrogen peroxide (H_2_O_2_). It was expressed as nmoles/mg protein^[^^16^^]^. NO in aortic samples was examined by measuring the level of nitrite, an oxidative metabolite of endogenous NO, by using the Griess reagent, in which a chromophore with a strong absorbance at 542 nm is formed by reaction of nitrite with a mixture of naphthyletilenediamine (0.1%) and sulphanilamide (1%). The absorbance was measured in a spectrophotometer to give the nitrite concentration^[^^17^^]^. Protein was measured by the method of Lowry et al.^[^^18^^]^, using bovine serum albumin as standard.

### Statistical Analysis

All data are expressed as the mean ± standard deviation. Comparisons between two groups were performed by Fisher's exact test and statistical analysis for three groups included the ANOVA method followed by t test. A statistical significance was assumed to be α=5%.

## RESULTS

Twenty-six patients were divided into three groups: control group (n=10) formed by cadaveric organ donors (aged 22 to 51 years old); SAS group (n=9) formed by patients with SAS; and TAO group (n=7) formed by patients with chronic total aortoiliac occlusion. The clinical and surgical data are summarized in Table 1. In each group, there were 4 women. The mean age, body mass index (BMI), and left ventricular ejection fraction (LVEF) were similar between the SAS and TAO groups. In relation to surgical data, there was no perioperative death in both groups. The duration of the procedures, blood loss, and intensive care unit (ICU) stay as well as postoperative hospital stay were also similar between the SAS and TAO groups (Table 1).

**Table 1:** Clinical characteristics and surgical data of patients with severe aortoiliac stenosis (SAS) and totally aortoiliac occlusion (TAO) submitted to aortobifemoral bypass.

**Characteristics**	**SAS group (n=9)**	**TAO group (n=7)**	**P values**
Mean age (years)	57.7±5.86	55.1±5.91	0.42
Women n (%)	4 (44.5)	4 (57.1)	1.00
Tobacco use (%)	9 (100)	7 (100)	1.00
Hypertension (%)	8 (88.9)	5 (71.4)	0.53
Hyperlipidemia (%)	3 (33.4)	2 (28.6)	1.00
Diabetes (%)	2 (22.3)	-	0.47
BMI (kg/m^2^)	25.8±3.25	21.8±4.33	0.13
LVEF (%)	60±2.67	67.5±8.01	0.06
Systolic pressure (mmHg)	131.14±9.96	129.3±14.24	0.79
Diastolic pressure (mmHg)	60.7±8.3	71.7±15.7	0.19
Creatinine (mg/dL)	0.92±0.14	0.94±0.08	0.88
Perioperative death	0	0	1.00
Duration of procedures (minutes)	269.2±54.34	228±44.9	0.25
Blood loss (mL)	637.5±316.9	614.3±203.3	0.87
Median postoperative days (range)	10.5 (7-14)	11 (6-19)	0.56
Median ITU stay in hours (range)	46 (28-72)	40 (30-58)	0.17
Lower preoperative ABI	0.44±0.13	0.39±0.08	0.48
Postoperative ABI	0.78±0.12	0.84±0.17	0.47

ABI=ankle-brachial index; BMI=body mass index; LVEF=left ventricular ejection fraction

**Fig. 1:**
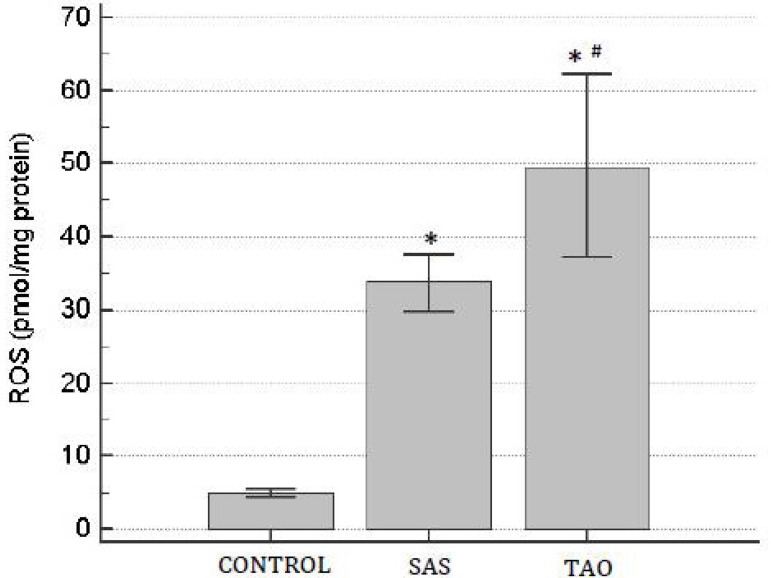
Levels of reactive oxygen species (ROS) on human aortas of patients with severe aortoiliac occlusive stenosis (SAS) and with total aortoiliac occlusion (TAO). Values are expressed in mean and the brackets represent interval of confidence of 95%. ANOVA followed test t. *P<0.05 when compared to control group; and ^#^P<0.05 when compared to SAS group.

The aortic ROS levels were significantly higher in the SAS and TAO groups when compared to the control group (*P*<0.05). Patients with TAO demonstrated higher levels of ROS (48.3±10.22 pmol/mg protein) when compared to the SAS group (33.02±4.54 pmol/mg protein; *P*<0.05) (Figure 1).

**Fig. 2:**
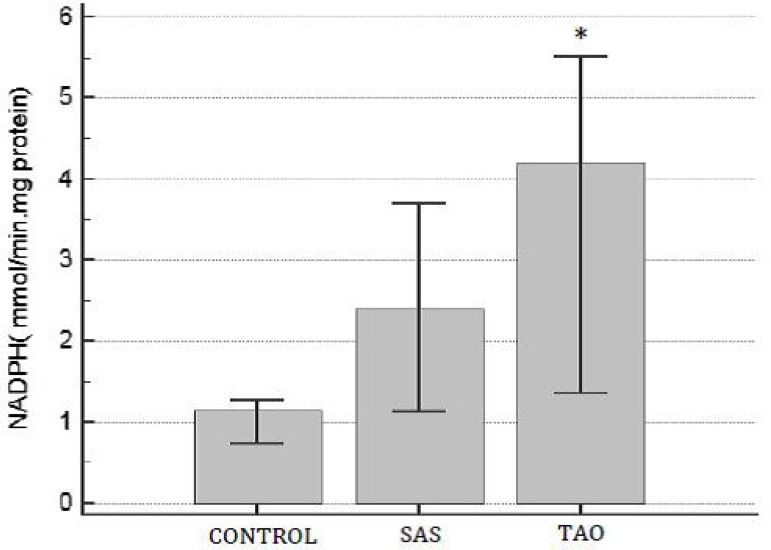
NADPH oxidase activity on human aortas of patients with severe aortoiliac occlusive stenosis (SAS) and with total aortoiliac occlusion (TAO). Values are expressed in mean and the brackets represent interval of confidence of 95%. ANOVA followed test t. *P<0.05 when compared to control group..

Measurement of NADPH oxidase activity demonstrated that TAO group had an increment when compared to control group (Figure 2). Moreover, there was no difference in the NADPH oxidase activity between SAS and others groups (*P*>0.05).

**Fig. 3:**
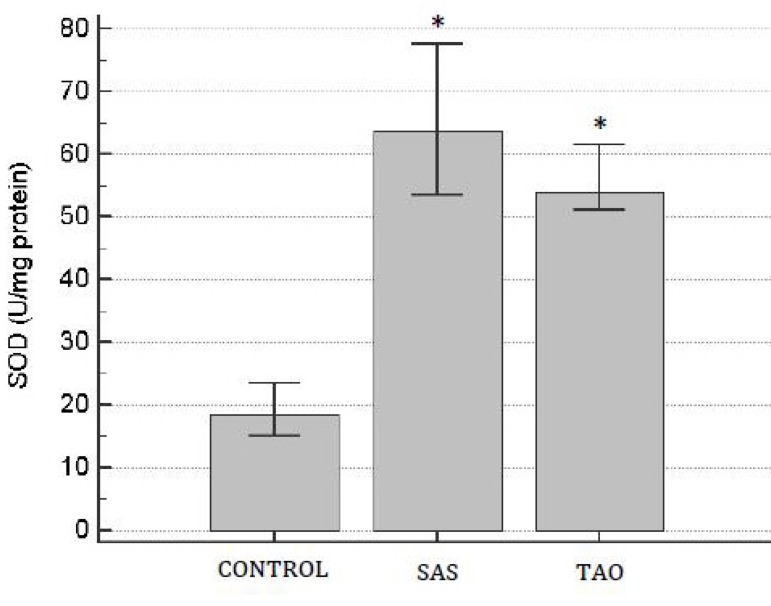
Superoxide dismutase (SOD) activity on human aortas of patients with severe aortoiliac occlusive stenosis (SAS) and with total aortoiliac occlusion (TAO). Values are expressed in mean and the brackets represent interval of confidence of 95%. ANOVA followed test t. *P<0.05 when compared to control group.

**Fig. 4:**
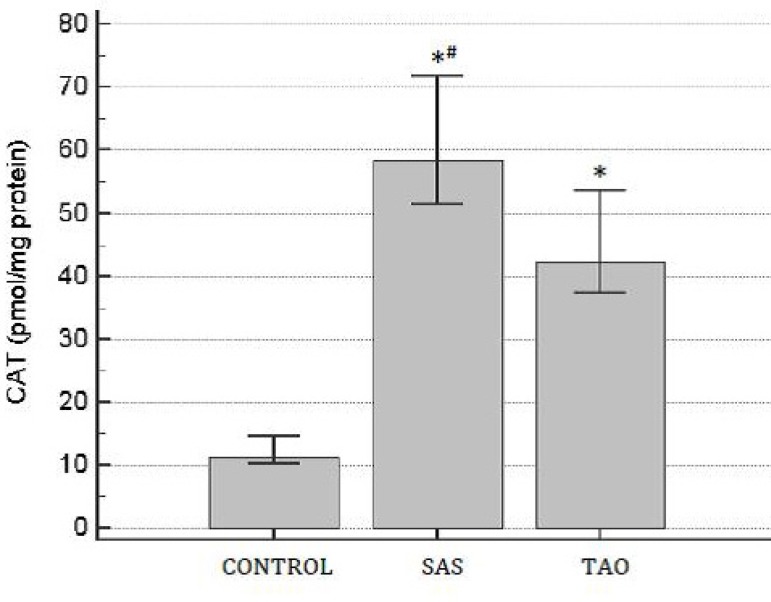
Catalase (CAT) activity on human aortas of patients with severe aortoiliac occlusive stenosis (SAS) and with total aortoiliac occlusion (TAO). Values are expressed in mean and the brackets represent interval of confidence of 95%. ANOVA followed test t. *P<0.05 when compared to control group; ^#^P<0.05 when compared to TAO group.

It was observed an increase of SOD activity in the SAS and TAO groups when compared to the control group (*P*<0.05) (Figure 3). However, there was no statistical difference between SAS and TAO groups. There was a significant increase of CAT activity in the SAS and TAO groups when compared to the control group. Furthermore, CAT activity in aortas from patients with SAS (60.2±9.5 pmol/mg protein) was statistically higher in comparing to the TAO group (44.74±7 pmol/mg protein; *P*<0.05) (Figure 4).

**Fig. 5:**
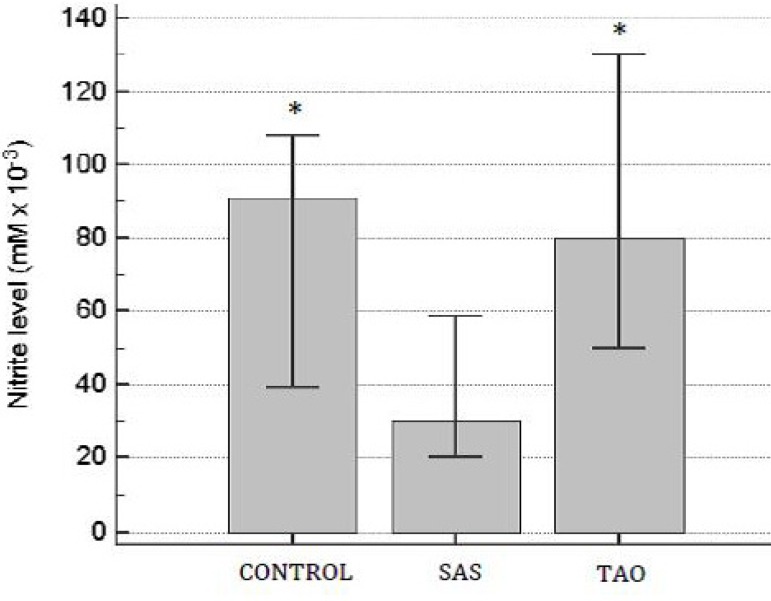
Nitrite levels on human aortas of patients with severe aortoiliac occlusive stenosis (SAS) and with total aortoiliac occlusion (TAO). Values are expressed in mean and the brackets represent interval of confidence of 95%. ANOVA followed test t. *P<0.05 when compared to SAS group.

Nitrite levels were significantly lower in the aortic samples of patients with SAS when compared to the other groups (*P*<0.05). Furthermore, there was no difference in the nitrite levels between TAO and the control group (*P*>0.05) (Figure 5).

## DISCUSSION

Advanced AOD tends to occur in relatively young patients who have a history of tobacco abuse. Basically, patients with SAS and TAO are treated by the aortobifemoral bypass and the surgical results in these patients seems to be similar^[^^2^^,^^19^^]^.

The major finding of our study was to demonstrate the role of oxidative stress in the atherosclerosis in patients with different degrees of AOD. Our results demonstrated that ROS levels were progressively higher as more severe as the AOD was. This way, patients with SAS or TAO had a higher ROS levels on their aorta samples; and the TAO group demonstrated a more elevated of ROS levels than SAS group. The role of ROS in the onset and progression of atherosclerotic damage in aortas has been described in animal models of different illnesses, being the ROS important mediators in the signaling pathways of inflammation and atherogenesis^[^^4^^,^^5^^]^. In atherosclerosis, ROS production can increment endothelial dysfunction, vascular smooth muscle cells proliferation and apoptosis, and inflammatory response^[^^20^^]^. Another markers of oxidative stress, such as levels of O_2_^-^, thiobarbituric acid and conjugated dienes were also evaluated in aortic samples of patients with atherosclerosis^[^^21^^]^.

Xiong et al.^[^^22^^]^ demonstrated that the earliest changes in an experimental model of AAA were associated with the local production of ROS. In the study of Miller et al.^[^^21^^]^, it was measured levels of O_2_^-^ and lipid peroxidation products in segments of AAA and in adjacent nonaneurysmal aortic tissue removed from patients undergoing elective AAA repair. The results indicated that levels of ROS are locally increased in AAA, partially because of NADPH oxidase activity, and lead to marked increases in oxidative stress. There are evidences that NADPH oxidase expression and activity are upregulated in atherosclerosis^[^^23^^]^. Analysis of non-atherosclerotic *versus* atherosclerotic human carotid arteries demonstrated a higher level of NADPH oxidase expression in atherosclerotic arteries^[^^23^^]^. We also observed an increase of NADPH oxidase activity in patients with TAO when compared to the control group. Haidari et al.^[^^3^^]^ observed an increase of oxidative stress in atherosclerosis-predisposed regions of the normal C57BL/6 mouse aorta, implicating NADPH oxidase as a possible contributing enzyme.

The imbalance between oxidants and antioxidants in favor of the former ones plays a significant role in the pathogenesis of atherosclerotic vascular disease^[^^6^^]^. Thus, knowledge of the activity of antioxidants enzymes is very important. Under normal conditions, SOD is the first line of defense against oxidative stress^[^^5^^]^. Dubick et al.^[^^24^^]^ demonstrated a lower activity of SOD in human aortic samples of patients with AAA and AOD in comparing to non-atherosclerotic organ donors. In another work, the same group of authors also compared the SOD activity between aortic specimens of patients with aneurysmal and occlusive disease. These authors evidenced a reduced SOD activity in occlusive (n=14) compared to aneurysmal (n=29) group^[^^25^^]^. In contrast, our study showed a significant increase of SOD activity in the SAS and TAO samples when compared to the control group, maybe because a compensative phenomenon to minimize oxidative stress. Furthermore, the role of oxidative stress in different aortopathies was evaluated by Soto et al.^[^^5^^]^. In this study, there was an increase in SOD activity in aorta specimens of patients with hypertension, Marfan and Turner's syndromes^[^^5^^]^.

Catalase is another important antioxidant enzyme, which is found in the liver, kidney, and aorta. Catalase uses two H_2_O_2_ molecules to break them into O_2_^-^; one acts as a reducing agent and the other as an oxidant agent. Overexpression of CAT prevents the stimulation of ROS and can prevent AAA formation in experimental setting^[^^26^^]^. Moreover, a diminished CAT expression and activity were observed in polymorphonuclear neutrophils in patients with AAA^[^^27^^]^. Soto et al.^[^^5^^]^ also showed that CAT activity increased in the patients with different aortopathies. Our results corroborate these findings that the increase of CAT activity in SAS and TAO groups may be due to overproduction of H_2_O_2_ in the aorta from these patients.

NO is usually quoted for its vasodilating properties and protective role in many vascular diseases and previous reports have indicated that decreased activity and production of NO play an important role in vascular disease^[^^11^^]^. Endothelial dysfunction and downregulated NO would contribute to the stiffness, reduced distensibility, and aortic complications such as AOD^[^^5^^]^. Our results showed a decrease of nitrite levels in SAS group may be due to endothelium dysfunction. Moreover, we did not observe an increase in nitrite levels in patients with TAO and this can be explained at least in part because of the higher levels of ROS which could increase the nitrite levels by inducible NOS activation, an enzyme which produces high quantities of NO, resulting in more oxidative stress^[^^11^^,^^21^^]^. Taking together, the higher levels of ROS and nitrite could facilitate intravascular thrombus formation by reducing the antiplatelets effects of NO^[^^28^^]^. Then, this aortic thrombus can provoke an inflammatory response in the aortic wall and can also limit the oxygen supply to endothelium resulting in increasing of NO production^[^^29^^]^. Liao et al.^[^^11^^]^ founded an overexpression of NOS which was positively correlated with the degree of inflammation in the aortic wall in patients with AAA.

The major limitation of this study is the small size of patients and aortic samples, preventing some correlation with clinical and angiographic findings. However, previous studies performed with human aortic samples have also been described with small number of samples and have obtained aortas from autopsy or organ donor patients as control group^[^^5^^,^^11^^,^^21^^,^^24^^,^^25^^]^. To our knowledge, this is the first study in which human aortic segments of patients with different degrees of AOD were directly compared.

## CONCLUSION

Our study on human aortic samples showed that oxidative stress seems to be related with the degree of AOD and the increase of antioxidant enzymes (SOD and CAT) activities may be due to a compensative phenomenon. Moreover, nitrite overproduction occurred in patients with chronic total aortic occlusion (TAO group) while its reduction was observed in severe aortoiliac stenosis (SAS group). Although antioxidant therapies did not have a consistent effect in some clinical trials for prevention atherosclerosis complications^[^^30^^]^, this preliminary study offer us a more comprehensive knowledge about the role of oxidative stress in atherosclerotic aortas in human beings.

**Table:** 

**Authors' roles & responsibilities**	
MLL	Analysis and/or data interpretation; conception and design study; manuscript redaction or critical review of its content; realization of operations and/or trials; statistical analysis; final manuscript approval
CCC	Analysis and/or data interpretation; final manuscript approval
ABK	Analysis and/or data interpretation; final manuscript approval
ANK	Manuscript redaction or critical review of its content; final manuscript approval
NA	Manuscript redaction or critical review of its content; final manuscript approval

## References

[1] Norgren L, Hiatt WR, Dormandy JA, Nehler MR, Harris KA, Fowkes FG, TASC II Working Group (2007). Inter-Society Consensus for the Management of Peripheral Arterial
Disease (TASC II). J Vasc Surg.

[2] Lucas ML, Deibler L, Erling N, Lichtenfels E, Aerts N (2015). Surgical treatment of chronic aortoiliac
occlusion. J Vasc Bras.

[3] Haidari M, Ali M, Gangehei L, Chen M, Zhang W, Cybulsky MI (2010). Increased oxidative stress in atherosclerosis-predisposed regions
of the mouse aorta. Life Sci.

[4] Roy Chowdhury SK, Sangle GV, Xie X, Stelmack GL, Halayko AJ, Shen GX (2010). Effects of extensively oxidized low-density lipoprotein on
mitochondrial function and reactive oxygen species in porcine aoric
endothelial cells. Am J Physiol Endocrinol Metab.

[5] Soto ME, Soria-Castro E, Lans VG, Ontiveros EM, Mejía BI, Hernandez HJ (2014). Analysis of oxidative stress enzymes and structural and
functional proteins on human aortic tissue from different
aortopathies. Oxid Med Cell Longev.

[6] Singh U, Jialal I (2006). Oxidative stress and atherosclerosis. Pathophysiology.

[7] Dias AE, Melnikov P, Cônsolo LZ (2015). Oxidative stress in coronary artery bypass
surgery. Rev Bras Cir Cardiovasc.

[8] Cao Y, Wang L, Chen H, Lv Z (2013). Beneficial effects of hyperosmotic perfusion in the myocardium
after ischemia/reperfusion injury in isolated rat hearts. Rev Bras Cir Cardiovasc.

[9] Pari L, Monisha P, Mohamed Jalaludeen A (2012). Beneficial role of diosgenin on oxidative stress in aorta of
streptozotocin induced diabetic rats. Eur J Pharmacol.

[10] Yajima N, Masuda M, Miyazaki M, Nakajima N, Chien S, Shyy JY (2002). Oxidative stress is involved in the development of experimental
abdominal aortic aneurysm: a study of the transcription profile with
complementary DNA microarray. J Vasc Surg.

[11] Liao MF, Jing ZP, Bao JM, Zhao ZQ, Mei ZJ, Lu QS (2006). Role of nitric oxide and inducible nitric oxide synthase in human
abdominal aortic aneurysms: a preliminary study. Chin Med J (Engl).

[12] Sartório CL, Fraccarollo D, Galuppo P, Leutke M, Ertl G, Stefanon I (2007). Mineralocorticoid receptor blockade improve vasomotor dysfunction
and vascular oxidative stress after myocardial infarction. Hypertension.

[13] LeBel CP, Ischiropoulos H, Bondy SC (1992). Evaluation of the probe 2',7'-dichlorofluorescin as an indicator
of reactive oxygen species formation and oxidative stress. Chem Res Toxicol.

[14] Wei Y, Sowers JR, Nistala R, Gong H, Uptergrove GM, Clark SE (2006). Angiotensin II-induced NADPH oxidase activation impairs insulin
signaling in skeletal muscle cells. J Biol Chem.

[15] Marklund SL (1985). Superoxide dismutase isoenzymes in tissues and plasma from New
Zealand black mice, nude mice and normal BALB/c mice. Mutat Res.

[16] Aebi H (1984). Catalase in vitro. Methods Enzymol.

[17] Granger DL, Anstey NM, Miller WC, Weinberg JB (1999). Measuring nitric oxide production in human clinical
studies. Methods Enzymol.

[18] Lowry OH, Rosebrough NJ, Farr AL, Randall RJ (1951). Protein measurement with the Folin phenol reagent. J Biol Chem.

[19] Chiu KW, Davies RS, Nightingale PG, Bradbury AW, Adam DJ (2010). Review of direct anatomical open surgical management of
atherosclerotic aorto-iliac occlusive disease. Eur J Endovasc Surg.

[20] Raaz U, Toh R, Maegdefessel L, Adam M, Nakagami F, Emrich FC (2014). Hemodynamic regulation of reactive oxygen species: implications
for vascular diseases. Antioxid Redox Signal.

[21] Miller FJ, Sharp WJ, Fang X, Oberley LW, Oberley TD, Weintraub NL (2002). Oxidative stress in human abdominal aortic aneurysms: a potential
mediator of aneurysmal remodeling. Arterioscler Thromb Vasc Biol.

[22] Xiong W, Mactaggart J, Knispel R, Worth J, Zhu Z, Li Y (2009). Inhibition of reactive oxygen species attenuates aneurysm
formation in a murine model. Atherosclerosis.

[23] Manea A, Manea SA, Gan AM, Constantin A, Fenyo IM, Raicu M (2015). Human monocytes and macrophages express NADPH oxidase 5; a
potential source of reactive oxygen species in
atherosclerosis. Biochem Biophys Res Commun.

[24] Dubick MA, Keen CL, DiSilvestro RA, Eskelson CD, Ireton J, Hunter GC (1999). Antioxidant enzyme activity in human abdominal aortic aneurysmal
and occlusive disease. Proc Soc Exp Biol Med.

[25] Dubick MA, Hunter GC, Casey SM, Keen CL (1987). Aortic ascorbic acid, trace elements, and superoxide dismutase in
human aneurysmal and occlusive disease. Proc Soc Exp Biol Med.

[26] Maiellaro-Rafferty K, Weiss D, Joseph G, Wan W, Gleason RL, Taylor WR (2011). Catalase overexpression in aortic smooth muscle prevents
pathological mechanical changes underlying abdominal aortic aneurysm
formation. Am J Physiol Heart Circ Physiol.

[27] Ramos-Mozo P, Madrigal-Matute J, Martinez-Pinna R, Blanco-Colio LM, Lopez JA, Camafeita E (2011). Proteomic analysis of polymorphonuclear neutrophils identifies
catalase as a novel biomaker of abdominal aortic aneurysm: potential
implication of oxidative stress in abdominal aortic aneurysm
progression. Arterioscler Thromb Vasc Biol.

[28] Ambrosio G, Tritto I, Golino P (1997). Reactive oxygen metabolites and arterial
thrombosis. Cardiovasc Res.

[29] Arnet UA, McMillan A, Dinerman JL, Ballermann B, Lowenstein CJ (1996). Regulation of endothelial nitric-oxide synthase during
hypoxia. J Biol Chem.

[30] Fearon IM, Faux SP (2009). Oxidative stress and cardiovascular disease: novel tools give
(free) radical insight. J Mol Cell Cardiol.

